# Poor reporting of multivariable prediction model studies: towards a targeted implementation strategy of the TRIPOD statement

**DOI:** 10.1186/s12916-018-1099-2

**Published:** 2018-07-19

**Authors:** Pauline Heus, Johanna A. A. G. Damen, Romin Pajouheshnia, Rob J. P. M. Scholten, Johannes B. Reitsma, Gary S. Collins, Douglas G. Altman, Karel G. M. Moons, Lotty Hooft

**Affiliations:** 1 Cochrane Netherlands, University Medical Center Utrecht, Utrecht University, Utrecht, The Netherlands; 2 Julius Center for Health Sciences and Primary Care, University Medical Center Utrecht, Utrecht University, Utrecht, The Netherlands; 3 Centre for Statistics in Medicine, NDORMS, Botnar Research Centre, University of Oxford, Oxford, UK

**Keywords:** TRIPOD, Reporting guideline, Prediction model, Risk score, Prediction rule, Risk assessment, Prognosis, Diagnosis, Development, Validation, Incremental value

## Abstract

**Background:**

As complete reporting is essential to judge the validity and applicability of multivariable prediction models, a guideline for the Transparent Reporting of a multivariable prediction model for Individual Prognosis Or Diagnosis (TRIPOD) was introduced. We assessed the completeness of reporting of prediction model studies published just before the introduction of the TRIPOD statement, to refine and tailor its implementation strategy.

**Methods:**

Within each of 37 clinical domains, 10 journals with the highest journal impact factor were selected. A PubMed search was performed to identify prediction model studies published before the launch of TRIPOD in these journals (May 2014). Eligible publications reported on the development or external validation of a multivariable prediction model (either diagnostic or prognostic) or on the incremental value of adding a predictor to an existing model.

**Results:**

We included 146 publications (84% prognostic), from which we assessed 170 models: 73 (43%) on model development, 43 (25%) on external validation, 33 (19%) on incremental value, and 21 (12%) on combined development and external validation of the same model. Overall, publications adhered to a median of 44% (25th–75th percentile 35–52%) of TRIPOD items, with 44% (35–53%) for prognostic and 41% (34–48%) for diagnostic models. TRIPOD items that were completely reported for less than 25% of the models concerned abstract (2%), title (5%), blinding of predictor assessment (6%), comparison of development and validation data (11%), model updating (14%), model performance (14%), model specification (17%), characteristics of participants (21%), model performance measures (methods) (21%), and model-building procedures (24%). Most often reported were TRIPOD items regarding overall interpretation (96%), source of data (95%), and risk groups (90%).

**Conclusions:**

More than half of the items considered essential for transparent reporting were not fully addressed in publications of multivariable prediction model studies. Essential information for using a model in individual risk prediction, i.e. model specifications and model performance, was incomplete for more than 80% of the models. Items that require improved reporting are title, abstract, and model-building procedures, as they are crucial for identification and external validation of prediction models.

**Electronic supplementary material:**

The online version of this article (10.1186/s12916-018-1099-2) contains supplementary material, which is available to authorized users.

## Background

Multivariable prediction models (risk scores or prediction rules) estimate an individual’s probability or risk that a specific disease or condition is present (diagnostic models) or that a specific event will occur in the future (prognostic models) based on multiple characteristics or pieces of information for that individual [1]. Such models are increasingly used by healthcare providers to support clinical decision making or to inform patients or relatives. Studies about prediction models may address the development of a new model, validation of an existing, previously developed model in other individuals (with or without adjusting or updating the model to the validation setting), or a combination of these two types [2–5]. Some prediction model studies evaluate the addition of a single predictor to an existing model (incremental value) [4].

In addition to appropriate design, conduct, and analysis, reporting of prediction model studies should be complete and accurate. Complete reporting of research facilitates study replication, assessment of the study validity (risk of bias), interpretation of the results, and judgment of applicability of the study results (e.g. the prediction model itself) to other individuals or settings. Clinicians and other stakeholders can only use previously developed and validated prediction models when all relevant information is available for calculating predicted risks at an individual level. High-quality information about prediction model studies is therefore essential.

Previous systematic reviews showed that within different clinical domains the quality of reporting of prediction models is suboptimal [6–11]. To improve the reporting of studies of prediction models, a guideline for the Transparent Reporting of a multivariable prediction model for Individual Prognosis Or Diagnosis (TRIPOD) was launched in January 2015 in more than 10 medical journals [12, 13]. The TRIPOD statement is a checklist of 22 items considered essential for informative reporting of prediction model studies. Both diagnostic and prognostic prediction model studies are covered by the TRIPOD statement, and the checklist can be used for all types of prediction model studies (development, external validation, and incremental value) within all clinical domains.

In this comprehensive literature review, we assessed the completeness of reporting of prediction model studies that were published just before the introduction of the TRIPOD statement. Our results provide key clues to further refine and tailor the implementation strategy of the TRIPOD statement.

## Methods

### Identification of prediction model studies

To cover a wide range of clinical domains, we started with 37 subject categories (2012 Journal Citation Reports®) [14] from which we selected the 10 journals with the highest journal impact factor (Additional file 1). After deduplication, 341 unique journals remained. We performed a search in PubMed to identify prediction model studies published in these journals before the launch of TRIPOD (May 2014), using a validated search filter for identifying prognostic and diagnostic prediction studies (Additional file 2) [15].

Eligible publications described the development or external validation of a multivariable prediction model (either diagnostic or prognostic) or evaluated the incremental value of adding a predictor to an existing model [1–5, 16]. We excluded so-called prognostic factor or predictor finding studies, as well as studies evaluating the impact of the use of a prediction model on management or patient outcomes [3, 7, 17]. We excluded prediction model studies using non-regression techniques (e.g. classification trees, neural networks, and machine learning) or pharmacokinetic models. Titles and abstracts of the retrieved publications were screened by one of two authors (JAAGD or PH). After reading the full text report, they judged whether to include or exclude a potentially eligible publication. Any doubts regarding definitive eligibility were discussed, if necessary, with a third author. If we were not able to retrieve the full text of a publication via our institutions, it was excluded.

### Data extraction

For each included publication we recorded the journal impact factor (2012 Journal Citation Reports®) [14], clinical domain, and whether the purpose of prediction was diagnostic or prognostic. Furthermore, we classified publications into four types of prediction model studies: development, external validation, incremental value, or combination of development and external validation of the same model. A publication could be categorised as more than one type of prediction model study. For example, if a publication reported on both development and external validation, but of different models, it was classified as development as well as external validation. If a publication included multiple prediction model studies of the same type, e.g. if two models were developed, we extracted data for only one model. If there was no primary model, we used the model that was studied in the largest sample. Information about study design, sample size, number of predictors in the final model, and predicted outcome was extracted for all included prediction models.

To judge the completeness of the reporting, we transformed items of the TRIPOD statement (Box 1) into a data extraction form, which was piloted extensively to ensure consistent extraction of the data. The TRIPOD statement consists of 22 main items, 10 of which are divided in two (items 3, 4, 6, 7, 14, 15, and 19), three (items 5 and 13), or five (item 10) subitems [12, 13]. For TRIPOD items (main or subitems, hereafter just called items) containing multiple reporting elements, we extracted information regarding each of these elements. For example, for item 4b, ’Specify the key study dates, including start of accrual, end of accrual, and, if applicable, end of follow-up’, we used three data extraction elements to record information regarding (1) the start of accrual, (2) end of accrual, and (3) end of follow-up. The data extraction form including all data extraction elements can be found on the website of the TRIPOD statement (www.tripod-statement.org/).

For each data extraction element we judged whether the requested information was available in the publication. If a publication reported both the development and external validation of the same prediction model, we extracted data on the reporting of either separately, and subsequently combined the extracted information for each data extraction element.

Three authors extracted data (JAAGD, PH, RP). If the authors disagreed or were unsure about the reporting of a data extraction element, it was discussed in consensus meetings with the other co-authors.

### Analyses

Based on the extracted data elements, we first determined whether the reporting of each TRIPOD item was complete (completeness is defined in the following subsection). We then calculated overall scores for completeness of reporting per model, per publication, and per item of the TRIPOD statement (across models).

#### Completeness of reporting of each TRIPOD item

The reporting of a TRIPOD item was judged to be complete if the requested information for all elements of that particular TRIPOD item was present. For elements belonging to TRIPOD items 4b, 5a, 6a, and 7a, we considered a reference to information in another article acceptable. If an element was not applicable to a specific model (e.g. follow-up might be not relevant in a diagnostic prediction model study) (item 4b), or blinding was a non-issue (e.g. if the predicted outcome was for example overall mortality) (items 6b and 7b), this element was regarded as being reported.

#### Overall completeness of reporting per model

To calculate the overall completeness of reporting for each included model, we divided the number of completely reported TRIPOD items by the total number of TRIPOD items for that model. The total number of TRIPOD items varies per type of prediction model study, as six of the TRIPOD items only apply to development of a prediction model (10a, 10b, 14a, 14b, 15a, and 15b) and six only to external validation (10c, 10e, 12, 13c, 17, and 19a). This resulted in a total number of 31 TRIPOD items for the reporting of either development or external validation of a prediction model, 37 for the combined reporting of development and external validation of the same prediction model, and 36 for reporting incremental value.

Five items of the TRIPOD statement include an ‘if done’ or ‘if applicable’ statement (items 5c, 10e, 11, 14b, and 17). If we considered such an item not applicable for a particular study, it was excluded when calculating the completeness of reporting (in both the numerator and denominator). Furthermore, item 21 of the TRIPOD statement was excluded from all calculations, as it refers to whether supplementary material was provided.

#### Overall completeness of reporting per publication

The overall reporting per publication equals the reporting per model (see previous subsection) for publications classified as development, external validation, incremental value, or combined development and external validation of the same model. For publications classified as more than one type of prediction model study, e.g. development of a model and external validation of a different model, we combined the reporting of the different prediction model types within that publication. Reporting was considered complete when the reporting of the different types of prediction model studies was complete, except for TRIPOD items 3a and 18–20, for which complete reporting for either type was considered sufficient.

We used linear regression to investigate possible relationships between completeness of reporting per publication as dependent variable, and sample size, journal impact factor, number of predictors in the final model, and prospective study design (as dichotomous variable, yes/no) as independent variables.

#### Overall completeness of reporting per item of the TRIPOD statement

We assessed the overall completeness of reporting of individual items of the TRIPOD statement by dividing the number of models with complete reporting of a particular TRIPOD item by the total number of models in which that item was applicable.

## Results

**Fig. 1:**
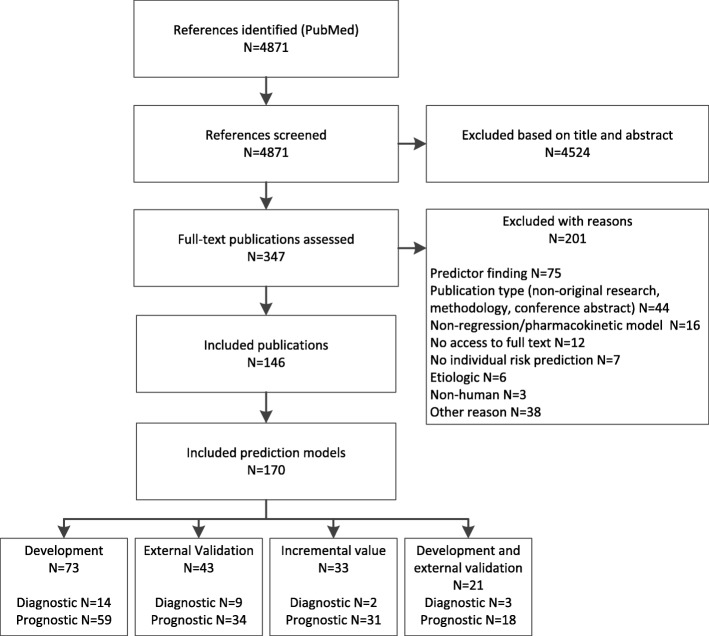
Flow diagram of selection procedure

We included a total of 146 publications (Fig. 1). Most publications (122 [84%]) reported prognostic models. From the 146 publications we scored the reporting of 170 prediction models: 73 (43%) concerned model development, 43 (25%) external validation of an existing model, 33 (19%) incremental value of adding a predictor to a model, and 21 (12%) a combination of development and external validation of the same model.

The three clinical domains with the most publications of prediction models were critical care medicine (18 [11%]), obstetrics and gynaecology (15 [9%]), and gastroenterology and hepatology (12 [7%]). The median journal impact factor of the publications was 5.3 (25th–75th percentile [*P*_25_–*P*_75_] 4.0–7.1). The median sample size of the populations in which a model was studied was 450 (*P*_25_–*P*_75_ 200–2005). In the final models a median of 5 (*P*_25_–*P*_75_ 3–8) predictors were included, and in 23 models (16%) all-cause mortality was the predicted outcome.

### Completeness of reporting per publication

**Fig. 2:**
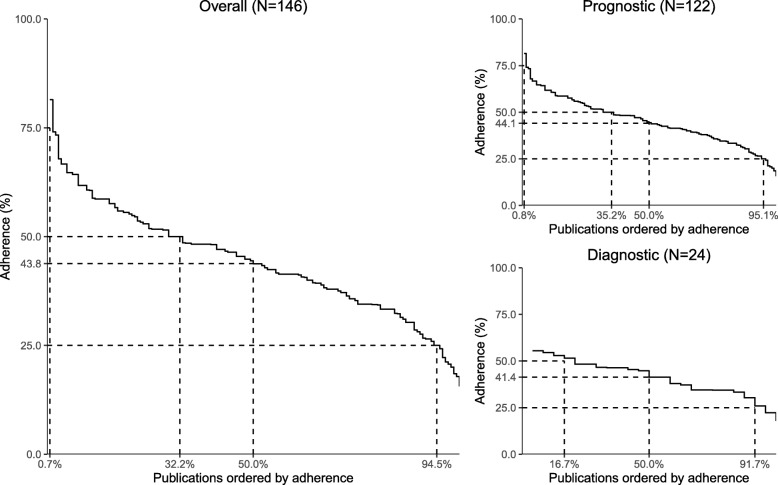
Reporting across publications: adherence to items of the TRIPOD statement

Overall, publications adhered to between 16 and 81% of the items of the TRIPOD statement with a median of 44% (*P*_25_–*P*_75_ 35–52%) (Fig. 2). The reporting quality for prognostic and diagnostic prediction models was comparable, with a median adherence of 44% (*P*_25_–*P*_75_ 35–53%) and 41% (*P*_25_–*P*_75_ 34–48%), respectively. The most complete reporting was seen for the combined reporting of development and external validation of the same model (47%; *P*_25_–P_75_ 35–54%), followed by the reporting of model development (43%; *P*_25_–*P*_75_ 35–53%), external validation (43%; *P*_25_–*P*_75_ 37–54%), and incremental value (38%; *P*_25_–*P*_75_ 33–49%). No associations were found between completeness of reporting and sample size, journal impact factor, number of predictors in the final model, and prospective study design (data not shown).

### Reporting of individual TRIPOD items

Six TRIPOD items were reported in 75% or more of the 170 models, and 10 items in less than 25% (Table 1).

**Table 1:** Completeness of reporting of individual TRIPOD items (*n* = 170 models)

Complete reporting for > 75% of the models			Complete reporting for < 25% of the models		
TRIPOD items		%	TRIPOD items		%
19b	Give an overall interpretation of the results, considering objectives, limitations, results from similar studies, and other relevant evidence	96	10b	Specify type of model, all model-building procedures (including any predictor selection), and method for internal validation	24
4a	Describe the study design or source of data (e.g. randomised trial, cohort, or registry data), separately for the development and validation data sets, if applicable	95	10d	Specify all measures used to assess model performance and, if relevant, to compare multiple models	21
11	Provide details on how risk groups were created, if done	90	13b	Describe the characteristics of the participants (basic demographics, clinical features, available predictors), including the number of participants with missing data for predictors and outcome	21
18	Discuss any limitations of the study (such as non-representative sample, few events per predictor, missing data)	88	15a	Present the full prediction model to allow predictions for individuals (i.e. all regression coefficients, and model intercept or baseline survival at a given time point)	17
3a	Explain the medical context (including whether diagnostic or prognostic) and rationale for developing or validating the multivariable prediction model, including references to existing models	81	16	Report performance measures (with confidence intervals [CIs]) for the prediction model	14
5b	Describe eligibility criteria for participants	79	17	If done, report the results from any model updating (i.e. model specification, model performance)	14
			12	For validation, identify any differences from the development data in setting, eligibility criteria, outcome, and predictors	11
			7b	Report any actions to blind assessment of predictors for the outcome and other predictors	6
			1	Identify the study as developing and/or validating a multivariable prediction model, the target population, and the outcome to be predicted	5
			2	Provide a summary of objectives, study design, setting, participants, sample size, predictors, outcome, statistical analysis, results, and conclusions	2

**Fig. 3:**
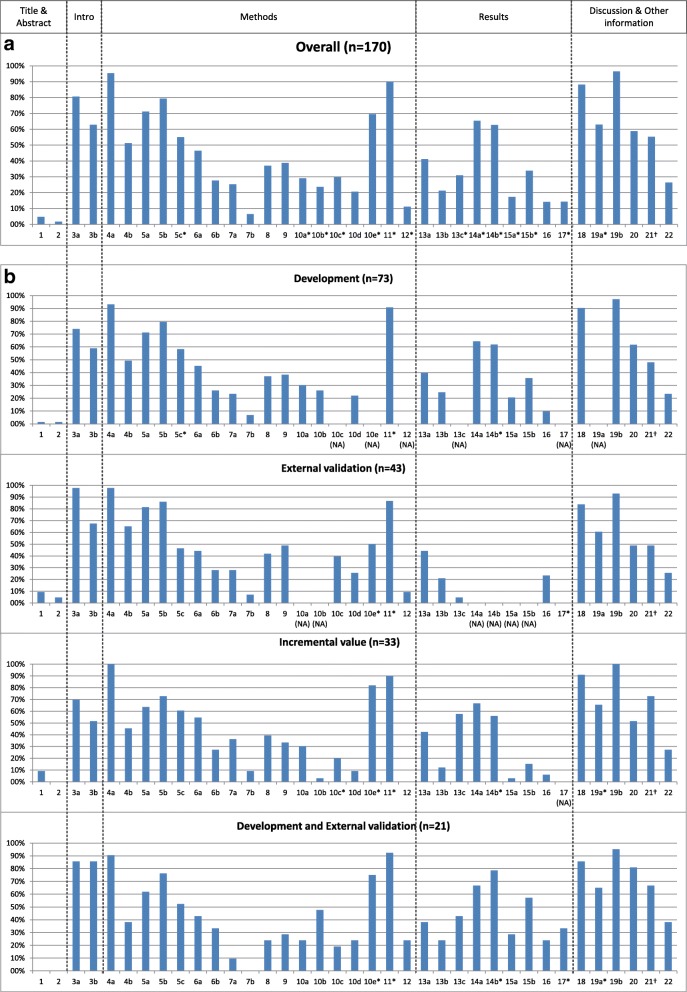
Reporting of the items of the TRIPOD statement overall (**a**) and per type of prediction model study (**b**) (see Box 1 for list of items of the TRIPOD statement). *NA* not applicable (not all items of the TRIPOD statement are relevant to all types of prediction model studies). Percentages are based on number of models for which an item was applicable (and thus should have been reported). *Where this number deviates from the total number of models, this is indicated. This concerns the following items (*N* = number of models for which the item was applicable). Overall: 5c (*N* = 169), 10a (*N* = 127), 10b (*N* = 127), 10c (*N* = 84), 10e (*N* = 23), 11 (*N* = 70), 12 (*N* = 81), 13c (*N* = 97), 14a (*N* = 127), 14b (*N* = 94), 15a (*N* = 127), 15b (*N* = 127), 17 (*N* = 7), 19a (*N* = 92); Development: 5c (*N* = 72), 11 (*N* = 22), 14b (*N* = 55); External validation: 10e (*N* = 8), 11 (*N* = 15), 17 (*N* = 4); Incremental value: 10c (*N* = 20), 10e (*N* = 11), 11 (*N* = 20), 12 (*N* = 17), 14b (*N* = 25), 19a (*N* = 29); Development and external validation: 10e (*N* = 4), 11 (*N* = 13), 14b (*N* = 14), 17 (*N* = 3), 19a (*N* = 20). †Item 21 ’Provide information about the availability of supplementary resources, such as study protocol, Web calculator, and data sets’: the number of models for which this item was applicable is unknown. It probably was applicable to all models that reported this item. Instead of presenting a percentage of 100, we based the percentage on the total number of models.

Completeness of reporting of individual TRIPOD items is presented in Fig. 3 and Additional file 3 over all 170 models, and per type of prediction model study. The most notable findings for each section of the TRIPOD statement (title and abstract, introduction, methods, results, discussion, and other information) are described below.

#### Title and abstract (items 1 and 2)

According to the TRIPOD statement, an informative title contains (synonyms for) the term *risk prediction model*, the *type of prediction model* study (i.e. development, external validation, incremental value, or combination), the *target population,* and *outcome to be predicted*. Eight of the 170 models (5%) addressed all four elements. The description of the type of prediction model study was the least reported element (12%). Complete reporting of abstracts required information for 12 elements. Three of the models (2%) fulfilled all the requirements.

#### Introduction (item 3)

For 81% of the models complete information about background and rationale was provided (item 3a), and in 63% reporting of study objectives (item 3b), including a specification of the type of prediction model study, was considered complete.

#### Methods (items 4–12)

Source of data (item 4a; 95% reported) and eligibility criteria (item 5b; 79%) were among the best reported items for all four types of prediction model studies. Actions to blind assessment of (non-objective) outcomes (item 6b; 28%) and predictors (item 7b; 7%) were less well reported. Detailed predictor definitions (item 7a) were provided for 25% of the models. Also, information about how missing data were handled (item 9) was incomplete for the majority of models (reported in 39%). Most aspects of statistical analysis were inadequately reported as well. How predictors were handled (item 10a) was described in 29% of the models. Model-building procedures (item 10b) were specified in 24% overall, and were particularly poorly represented in incremental value reports (3%). Few studies (21%) described both discrimination and calibration as measures of model performance (item 10d).

#### Results (items 13–17)

Characteristics of participants (item 13b, complete reporting in 21%) were often reported without information regarding missing data for predictors and outcome. Two (5%) of the external validations presented demographics, distribution of predictors, and outcomes alongside those of the original development study (item 13c), and in combined reports of development and external validation this was done in 43%. The final model was presented in full (item 15a) in 17% of the models. For many models the intercept (or the cumulative baseline hazard, or baseline survival, for at least one time point in the case of survival models) was not provided. A small number of models provided information on both discrimination and calibration when reporting model performance (item 16; 14%). Discrimination was more frequently reported (79%) than calibration (29%).

#### Discussion (items 18–20)

An overall interpretation of the results (item 19b) was given for almost all included models of all types of prediction model studies (97%). The potential for clinical use and implications for future research (item 20) were discussed in 59% of the models.

#### Other information (items 21 and 22)

Information about the availability of supplementary resources (item 21) was provided in 55% of the models. Complete information regarding funding (item 22) was reported in 27%.

## Discussion

Complete and accurate reporting of prediction model studies is required to critically appraise, externally validate, evaluate the impact of, and eventually use prediction models in clinical practice. Our study shows that, regardless of the type of prediction model study and whether diagnostic or prognostic, more than half of the items deemed essential to report in prediction model publications according to the TRIPOD statement were not completely reported.

Highly problematic TRIPOD items in terms of reporting were items regarding title and abstract. These items, for which complete reporting requires information on multiple elements, were adequately reported for less than 10% of the models. In addition, details of study methods, especially blinding of outcome and predictor assessments, were provided for only a minority of reported models. Furthermore, information on follow-up, predictor definitions, model-building procedures, and handling of missing data were often lacking. Notable findings regarding the reporting of study results were that in more than 70% of the included models the final model was not presented in enough detail to make predictions for new patients, and that the reporting of model performance was often incomplete. Items of the TRIPOD statement that were generally well reported addressed the source of data and eligibility criteria, risk groups (if applicable), study limitations, and overall interpretation of results.

### Comparison with other studies

Our main finding of inadequate reporting in the majority of publications within 37 clinical domains is comparable to the findings of systematic reviews of prediction model studies performed in general medicine or specific clinical domains [6–11]. Inadequate reporting is considered to be a form of research waste [18, 19]. Therefore, for many study types, reporting guidelines were published in the last 20 years, such as the Consolidated Standards of Reporting Trials (CONSORT) statement in 1996 (updates in 2001 and 2010), the Standards for Reporting of Diagnostic Accuracy (STARD) statement in 2003 (update in 2015), and Reporting recommendations for tumour marker prognostic studies (REMARK) in 2005 [20–24]. Completeness of reporting before the introduction of these reporting guidelines was similar to our result of 44% adherence. Moher and colleagues (2001) evaluated 97 reports of randomised trials before the introduction of CONSORT and found adequate reporting for just over half of the items (58%) [25]. In a systematic review of 16 studies evaluating the adherence to STARD, overall, 51% of items were adequately reported [26]. For six included studies with quantitative data before publication of STARD, a range of 44–61% adherence was reported. An assessment of the reporting of prognostic studies of tumour markers was done shortly after the introduction of REMARK [27, 28]. Ten (out of 20) items were evaluated, and, overall, articles adhered to 53% of these.

### Strengths and limitations of this study

With this literature review we cover a broad literature base by including three major types of prediction model studies, both prognostic and diagnostic, across 37 clinical domains. Despite the use of a validated search strategy, we may have missed publications on prediction models. It is likely that the completeness of reporting of prediction models in these studies would have been worse. Furthermore, we selected studies from high impact journals. Therefore, our results on the completeness of reporting might be an optimistic representation of the reporting of prediction model studies in general.

In accordance with the TRIPOD statement, we included prediction models based on regression modelling approaches [13]. Although most TRIPOD items would apply, transparent reporting of prediction models using non-regression modelling techniques may require additional details, especially regarding model-building procedures, and specific guidance might be desirable.

We were strict in scoring adherence by requiring complete information on all elements of a TRIPOD item; e.g. complete reporting of model performance required the provision of both discrimination and calibration measures. This is in line with the nature of TRIPOD as having essential items needed to appraise and utilise a prediction model. However, authors might have good reasons not to provide specific details regarding an item. For example, if they believe that their model should not be validated or used in clinical practice, they may have decided not to present the coefficients of the full model. In the current study we would have scored TRIPOD item 15a as ’incompletely reported’. Although strict scoring potentially leads to poorer adherence results, it is needed for reasons of consistency.

We used two different denominators in our analyses, the number of publications (*n* = 146) and the number of models (*n* = 170), which implies that in the ’model’ analysis a number of publications were included multiple times. It is likely that results from the same publication, although based on the reporting of different models, are correlated. Given the descriptive nature of our analysis, we did not adjust for such a possible correlation.

We present results from studies that were published 4 years ago; nevertheless, we expect these findings to still be applicable and relevant to current publications of prediction models. From evaluations of other reporting guidelines, like CONSORT and STARD, we know that it takes time to demonstrate the impact of a reporting guideline on completeness of reporting, and changes over several years might be small [25, 26, 28–33]. In our opinion, therefore, it is too early for a before-after comparison at this moment, and the focus should first be on optimal implementation of TRIPOD.

### Implications for practice and areas for future research

Inadequate reporting impedes the use of all available evidence regarding a prediction model. First, as title and abstract were among the least well-reported items, identifying publications of prediction model studies might be challenging. In addition, we found the reporting of model development often insufficiently detailed, which makes external validation almost impossible. As a consequence, a new model might be developed, rather than making use of an existing model. Also, without model specifications it is impossible to use the model in clinical practice. Finally, inadequate reporting hinders critical appraisal and, thereby, the possibility of methodological investigation of sources of variation and bias in prediction model studies.

Experiences from other research areas indicate that the improvement in reporting after the introduction of a guideline is often slow and might be subtle [25, 26, 28–33]. Improving the completeness of reporting of prediction models is probably even more challenging, as it is a relatively young, less well-known research field, with methodology still in development and not yet strongly embedded in education. Moreover, the multivariable nature of prediction model studies and their focus on absolute probabilities rather than on comparative measures require the reporting of many details on methods and results. In addition, practical issues, like word limits or journal requirements, could act as barriers for complete reporting.

The introduction of the TRIPOD statement was the first step in improving the reporting of prediction model studies. However, more activities should be undertaken to enhance the implementation of the TRIPOD statement. Active implementation involves a collaborative effort of developers of a reporting guideline and other stakeholders within the academic community, like journal editors and educational institutions. Apart from raising awareness and providing training, possible post-publication activities that are recommended are encouraging guideline endorsement, asking for feedback, and evaluating the impact of the reporting guideline [34].

By highlighting the flaws in the reporting of prediction model studies, our results enable a targeted implementation strategy for the TRIPOD statement. Possible future activities are the development of educational materials and training regarding specific aspects of the reporting of prediction model studies. The examples of both adequate and suboptimal reporting within our data set can be used in the training of different stakeholders. An initiative that already has been started by the TRIPOD Group is the development of specific guidance on informative reporting of prediction model studies in abstracts [35]. Furthermore, as TRIPOD is periodically being reappraised and will be updated if necessary, our study will provide useful input for modifications of specific TRIPOD items, related to content, phrasing, or more detailed explanation [12]. Finally, our study will serve as a baseline measurement for future studies evaluating the impact of the introduction of the TRIPOD statement.

## Conclusions

Prediction models are poorly reported: more than half of the items that are considered essential for transparent reporting of a prediction model were not or were inadequately reported, especially with regard to details of the title, abstract, blinding, model-building procedures, the final model, and model performance. The results of this study can be used to further develop and refine the implementation and increase the impact of the TRIPOD statement.

## Box 1 Items of the TRIPOD statement

**Table:** 

*** Title and abstract***	
1. **Title (D; V)**: Identify the study as developing and/or validating a multivariable prediction model, the target population, and the outcome to be predicted	
2. **Abstract (D; V)**: Provide a summary of objectives, study design, setting, participants, sample size, predictors, outcome, statistical analysis, results, and conclusions	
*** Introduction***	
3. **Background and objectives**:	
a. **(D; V)** Explain the medical context (including whether diagnostic or prognostic) and rationale for developing or validating the multivariable prediction model, including references to existing models	
b. **(D; V)** Specify the objectives, including whether the study describes the development or validation of the model or both	
*** Methods***	
4. **Source of data**:	
a. **(D; V)** Describe the study design or source of data (e.g. randomised trial, cohort, or registry data), separately for the development and validation data sets, if applicable	
b. **(D; V)** Specify the key study dates, including start of accrual, end of accrual, and, if applicable, end of follow-up	
5. **Participants**:	
a. **(D; V)** Specify key elements of the study setting (e.g. primary care, secondary care, general population) including number and location of centres	
b. **(D; V)** Describe eligibility criteria for participants	
c. **(D; V)** Give details of treatments received, if relevant	
6. **Outcome**:	
a. **(D; V)** Clearly define the outcome that is predicted by the prediction model, including how and when assessed	
b. **(D; V)** Report any actions to blind assessment of the outcome to be predicted	
7. **Predictors**:	
a. **(D; V)** Clearly define all predictors used in developing or validating the multivariable prediction model, including how and when they were measured	
b. **(D; V)** Report any actions to blind assessment of predictors for the outcome and other predictors	
8. **Sample size (D; V)**: Explain how the study size was arrived at	
9. **Missing data (D; V)**: Describe how missing data were handled (e.g. complete-case analysis, single imputation, multiple imputation) with details of any imputation method	
10. **Statistical analysis methods**:	
a. **(D)** Describe how predictors were handled in the analyses	
b. **(D)** Specify type of model, all model-building procedures (including any predictor selection), and method for internal validation	
c. **(V)** For validation, describe how the predictions were calculated	
d. **(D; V)** Specify all measures used to assess model performance and, if relevant, to compare multiple models	
e. **(V)** Describe any model updating (e.g. recalibration) arising from the validation, if done	
11. **Risk groups (D; V)**: Provide details on how risk groups were created, if done	
12. **Development vs. validation (V)**: For validation, identify any differences from the development data in setting, eligibility criteria, outcome, and predictors	
*** Results***	
13. **Participants**:	
a. **(D; V)** Describe the flow of participants through the study, including the number of participants with and without the outcome and, if applicable, a summary of the follow-up time. A diagram may be helpful	
b. **(D; V)** Describe the characteristics of the participants (basic demographics, clinical features, available predictors), including the number of participants with missing data for predictors and outcome	
c. **(V)** For validation, show a comparison with the development data of the distribution of important variables (demographics, predictors, and outcome)	
14. **Model development**:	
a. **(D)** Specify the number of participants and outcome events in each analysis	
b. **(D)** If done, report the unadjusted association between each candidate predictor and outcome	
15. **Model specification**:	
a. **(D)** Present the full prediction model to allow predictions for individuals (i.e. all regression coefficients, and model intercept or baseline survival at a given time point)	
b. **(D)** Explain how to the use the prediction model	
16. **Model performance (D;V)**: Report performance measures (with confidence intervals [CIs]) for the prediction model	
17. **Model updating (V)**: If done, report the results from any model updating (i.e. model specification, model performance)	
*** Discussion***	
18. **Limitations (D;V)**: Discuss any limitations of the study (such as non-representative sample, few events per predictor, missing data)	
19. **Interpretation**:	
a. **(V)** For validation, discuss the results with reference to performance in the development data and any other validation data	
b. **(D;V)** Give an overall interpretation of the results, considering objectives, limitations, results from similar studies, and other relevant evidence	
20. **Implications (D;V)**: Discuss the potential clinical use of the model and implications for future research	
*** Other information***	
21. **Supplementary information (D;V)**: Provide information about the availability of supplementary resources, such as study protocol, Web calculator, and data sets	
22. **Funding (D;V)**: Give the source of funding and the role of the funders for the present study	

* D;V* item relevant to both development and external validation, *D* item only relevant to development, *V* item only relevant to external validation

## Additional files

Additional file 1: Journal selection. Ten journals with the highest journal impact factor within each of 37 categories (clinical domains) that were selected (2012 Journal Citation Reports® [Clarivate Analytics, 2017]). (PDF 430 kb)

Additional file 2: Search strategy. (PDF 268 kb)

Additional file 3: Reporting of the items of the TRIPOD statement. Completeness of reporting of the items of the TRIPOD statement, per type of prediction model study and overall. (PDF 492 kb)

## Abbreviations

CONSORT: CONsolidated Standards Of Reporting Trials
REMARK: REporting recommendations for tumour MARKer prognostic studies
STARD: STAndards for Reporting of Diagnostic accuracy
TRIPOD: Transparent Reporting of a multivariable prediction model for Individual Prognosis Or Diagnosis
