# Supplementary material for: Poor reporting of multivariable prediction model studies: towards a targeted implementation strategy of the TRIPOD statement
Source: BMC Med. 2018 Jul 19;16:120. doi: 10.1186/s12916-018-1099-2 (PMC6052616; doi:10.1186/s12916-018-1099-2)
Supplement: Supplementary file 1 — Journal selection. Ten journals with the highest journal impact factor within each of 37 categories (clinical domains) that were selected (2012 Journal Citation Reports® [Clarivate Analytics, 2017]). (PDF 430 kb) [file 12916_2018_1099_MOESM1_ESM.pdf]

Journal selection

Ten journals with the highest Journal Impact Factor within each of 37 categories (clinical domains) (2012 Journal Citation Reports® [Clarivate Analytics, 2017]) that were selected.
Full journal titles indicated with an * were included in more than one category.

Category (clinical domain)	Full journal title	Journal Impact Factor
Allergy	Journal of Allergy and Clinical Immunology*	12.047
	Allergy	5.883
	Clinical Reviews in Allergy & Immunology	5.590
	Clinical and Experimental Allergy	4.789
	Annals of Allergy Asthma & Immunology	3.449
	Current Opinion In Allergy and Clinical Immunology	3.398
	Pediatric Allergy and Immunology*	3.376
	Contact Dermatitis*	2.925
	Current Allergy and Asthma Reports	2.746
	Allergy Asthma & Immunology Research	2.653
Anesthesiology	Pain	5.644
	Anesthesiology	5.163
	British Journal of Anaesthesia	4.237
	Anaesthesia	3.486
	Regional Anesthesia and Pain Medicine	3.464
	Anesthesia and Analgesia	3.300
	European Journal of Pain	3.067
	Minerva Anestesiologica*	2.818
	European Journal of Anaesthesiology	2.792
	Pain Practice	2.605
Cardiac and cardiovasuclar systems	Circulation*	15.202
	European Heart Journal	14.097
	Journal of the American College of Cardiology	14.086
	Circulation Research*	11.861
	Nature Reviews Cardiology	10.400
	Circulation-Cardiovascular Genetics	6.728
	Circulation-Heart Failure	6.684
	Jacc-Cardiovascular Interventions	6.552
	Circulation-Cardiovascular Interventions	6.543
	Jacc-Cardiovascular Imaging*	6.164
Clinical neurology	Lancet Neurology	23.917
	Nature Reviews Neurology	15.518
	Alzheimers & Dementia	14.483
	Annals of Neurology	11.193
	Brain	9.915
	Acta Neuropathologica	9.734
	Sleep Medicine Reviews	8.681

	Neurology	8.249
	Archives of Neurology	7.685
	Neuro-Oncology	6.180
Critical care medicine	American Journal of Respiratory and Critical Care Medicine*	11.041
	Critical Care Medicine	6.124
	Chest*	5.854
	Intensive Care Medicine	5.258
	Critical Care	4.718
	Journal of Neurotrauma	4.295
	Resuscitation*	4.104
	Neurocritical Care	3.038
	Current Opinion In Critical Care	2.967
	Minerva Anestesiologica*	2.818
Dentistry. Oral surgery & medicine	Periodontology 2000	4.012
	Journal of Dental Research	3.826
	Clinical Implant Dentistry and Related Research	3.821
	Dental Materials	3.773
	Journal of Clinical Periodontology	3.688
	Clinical Oral Implants Research	3.433
	Journal of Dentistry	3.200
	Journal of Endodontics	2.929
	International Journal of Oral Science	2.719
	British Journal of Oral & Maxillofacial Surgery	2.717
Dermatology	Journal of Investigative Dermatology	6.193
	Pigment Cell & Melanoma Research	5.839
	Journal of the American Academy of Dermatology	4.906
	Archives of Dermatology	4.792
	British Journal of Dermatology	3.759
	Experimental Dermatology	3.578
	Journal of Dermatological Science	3.520
	Acta Dermato-Venereologica	3.487
	Contact Dermatitis*	2.925
	Skin Pharmacology and Physiology	2.885
Emergency medicine	Annals of Emergency Medicine	4.285
	Resuscitation*	4.104
	Emergencias	2.578
	Journal of Trauma-Injury Infection and Critical Care	2.348
	Injury-International Journal of the Care of the Injured	2.174
	Prehospital Emergency Care	1.859
	Academic Emergency Medicine	1.757
	American Journal of Emergency Medicine	1.704
	Scandinavian Journal of Trauma Resuscitation & Emergency Medicine	1.680
	Emergency Medicine Journal	1.645
Endocrinology & Metabolism	Endocrine Reviews	14.873

	Cell Metabolism	14.619
	Nature Reviews Endocrinology	11.025
	Trends In Endocrinology and Metabolism	8.901
	Frontiers In Neuroendocrinology	7.985
	Diabetes	7.895
	Diabetes Care	7.735
	Journal of Mammary Gland Biology and Neoplasia	7.524
	Journal of Pineal Research	7.304
	Antioxidants & Redox Signaling	7.189
Gastroenterology & Hepatology	Gastroenterology	12.821
	Hepatology	12.003
	Gut	10.732
	Nature Reviews Gastroenterology & Hepatology	10.426
	Journal of Hepatology	9.858
	Seminars In Liver Disease	8.274
	American Journal of Gastroenterology	7.553
	Clinical Gastroenterology and Hepatology	6.648
	Endoscopy*	5.735
	Gastrointestinal Endoscopy	5.210
Geriatrics & Gerontology	Neurobiology of Aging	6.166
	Ageing Research Reviews	5.953
	Aging Cell	5.705
	Journal of the American Medical Directors Association	5.302
	Frontiers In Aging Neuroscience	5.224
	Journals of Gerontology Series A-Biological Sciences and Medical Sciences	4.314
	American Journal of Geriatric Psychiatry	4.131
	Age	4.084
	Journal of the American Geriatrics Society	3.978
	Experimental Gerontology	3.911
Hematology	Circulation Research*	11.861
	Leukemia*	10.164
	Blood	9.060
	Stem Cells	7.701
	Arteriosclerosis Thrombosis and Vascular Biology*	6.338
	Thrombosis and Haemostasis*	6.094
	Journal of Thrombosis and Haemostasis*	6.081
	Blood Reviews	6.000
	Haematologica-the Hematology Journal	5.935
	Journal of Cerebral Blood Flow and Metabolism	5.398
Immunology	Annual Review of Immunology	36.556
	Nature Reviews Immunology	33.129
	Nature Immunology	26.199
	Immunity	19.795
	Journal of Experimental Medicine	13.214

	Immunological Reviews	12.155
	Journal of Allergy and Clinical Immunology*	12.047
	Trends In Immunology	9.486
	Clinical Infectious Diseases*	9.374
	Current Opinion In Immunology	8.771
Infectious diseases	Lancet Infectious Diseases	19.966
	Clinical Infectious Diseases*	9.374
	Aids	6.407
	Emerging Infectious Diseases	5.993
	Journal of Infectious Diseases	5.848
	Eurosurveillance	5.491
	Journal of Antimicrobial Chemotherapy	5.338
	Current Opinion In Infectious Diseases	4.870
	Current Opinion In Hiv and Aids	4.704
	Jaids-Journal of Acquired Immune Deficiency Syndromes	4.653
Integrative & complementary medicine	Alternative Medicine Review	4.857
	Phytomedicine	2.972
	Journal of Ethnopharmacology	2.755
	Integrative Cancer therapies	2.354
	American Journal of Chinese Medicine	2.281
	Complementary therapies In Medicine	2.093
	Bmc Complementary and Alternative Medicine	2.082
	Evidence-Based Complementary and Alternative Medicine	1.722
	Journal of Manipulative and Physiological therapeutics	1.647
	Journal of Alternative and Complementary Medicine	1.464
Medical laboratory technology	Clinical Chemistry	7.149
	Critical Reviews In Clinical Laboratory Sciences	3.783
	Advances In Clinical Chemistry	3.674
	Translational Research	3.490
	Clinical Chemistry and Laboratory Medicine	3.009
	Clinica Chimica Acta	2.850
	Archives of Pathology & Laboratory Medicine	2.781
	Clinical Biochemistry	2.450
	Therapeutic Drug Monitoring	2.234
	Cytometry Part B-Clinical Cytometry	2.231
Medicine. general & internal	New England Journal of Medicine	51.658
	Lancet	39.060
	Jama-Journal of the American Medical Association	29.978
	British Medical Journal	17.215
	Plos Medicine	15.253
	Annals of Internal Medicine	13.976
	Archives of Internal Medicine	10.579
	Bmc Medicine	6.679
	Canadian Medical Association Journal	6.465

	Journal of Internal Medicine	6.455
Obstetrics & Gynecology	Human Reproduction Update*	8.847
	Obstetrics and Gynecology	4.798
	Human Reproduction*	4.670
	Fertility and Sterility*	4.174
	Gynecologic Oncology	3.929
	American Journal of Obstetrics and Gynecology	3.877
	Bjog-An International Journal of Obstetrics and Gynaecology	3.760
	Ultrasound In Obstetrics & Gynecology	3.557
	Seminars In Reproductive Medicine*	3.211
	Menopause-the Journal of the North American Menopause Society	3.163
Oncology	Ca-A Cancer Journal For Clinicians	153.459
	Nature Reviews Cancer	35.000
	Lancet Oncology	25.117
	Cancer Cell	24.755
	Journal of Clinical Oncology	18.038
	Nature Reviews Clinical Oncology	15.031
	Jnci-Journal of the National Cancer Institute	14.336
	Leukemia*	10.164
	Cancer Discovery	10.143
	Biochimica Et Biophysica Acta-Reviews On Cancer	9.033
Ophthalmology	Progress In Retinal and Eye Research	9.439
	Ophthalmology	5.563
	Archives of Ophthalmology	3.826
	American Journal of Ophthalmology	3.631
	Investigative Ophthalmology & Visual Science	3.441
	Experimental Eye Research	3.026
	Survey of Ophthalmology	2.859
	Retina-the Journal of Retinal and Vitreous Diseases	2.825
	British Journal of Ophthalmology	2.725
	Ocular Surface	2.643
Orthopedics	American Journal of Sports Medicine*	4.439
	Osteoarthritis and Cartilage*	4.262
	Journal of Bone and Joint Surgery-American Volume	3.234
	Spine Journal	3.220
	Arthroscopy-the Journal of Arthroscopic and Related Surgery	3.103
	Journal of Orthopaedic & Sports Physical therapy*	2.947
	Journal of Orthopaedic Research	2.875
	Clinical Orthopaedics and Related Research	2.787
	Physical therapy*	2.778
	Acta Orthopaedica	2.736
Otorhinolaryngology	Ear and Hearing	3.262
	Jaro-Journal of the Association For Research In Otolaryngology	2.952
	Head and Neck-Journal For the Sciences and Specialties of the Head and Neck	2.833

	Hearing Research	2.537
	Audiology and Neuro-Otology	2.318
	Otology & Neurotology	2.014
	Laryngoscope	1.979
	Dysphagia	1.938
	Clinical Otolaryngology	1.869
	Archives of Otolaryngology-Head & Neck Surgery	1.779
Pediatrics	Journal of the American Academy of Child and Adolescent Psychiatry*	6.970
	Pediatrics	5.119
	Archives of Pediatrics & Adolescent Medicine	4.282
	Journal of Pediatrics	4.035
	European Child & Adolescent Psychiatry	3.699
	Pediatric Infectious Disease Journal	3.569
	Seminars In Fetal & Neonatal Medicine	3.505
	Archives of Disease In Childhood-Fetal and Neonatal Edition	3.451
	Pediatric Allergy and Immunology*	3.376
	Archives of Disease In Childhood	3.051
Peripheral vascular disease	Circulation*	15.202
	Circulation Research*	11.861
	Hypertension	6.873
	Arteriosclerosis Thrombosis and Vascular Biology*	6.338
	Stroke	6.158
	Thrombosis and Haemostasis*	6.094
	Journal of Thrombosis and Haemostasis*	6.081
	Current Opinion In Lipidology	5.839
	Atherosclerosis Supplements	4.333
	Seminars In Thrombosis and Hemostasis	4.216
Primary health care	Annals of Family Medicine	4.613
	Primary Care Respiratory Journal	2.191
	British Journal of General Practice	2.034
	Scandinavian Journal of Primary Health Care	1.905
	Family Practice	1.828
	Canadian Family Physician	1.808
	Journal of the American Board of Family Medicine	1.758
	American Family Physician	1.611
	Bmc Family Practice	1.609
	Primary Care Diabetes	1.609
Psychiatry	Molecular Psychiatry	14.897
	American Journal of Psychiatry	14.721
	Archives of General Psychiatry	13.772
	Biological Psychiatry	9.247
	World Psychiatry	8.974
	Neuropsychopharmacology	8.678
	Schizophrenia Bulletin	8.486

	Psychotherapy and Psychosomatics	7.230
	Journal of the American Academy of Child and Adolescent Psychiatry*	6.970
	British Journal of Psychiatry	6.606
Public. Environmental and Occupational health	Epidemiologic Reviews	9.269
	Environmental Health Perspectives	7.260
	International Journal of Epidemiology	6.982
	Who Technical Report Series	6.100
	Epidemiology	5.738
	Journal of Clinical Epidemiology	5.332
	Bulletin of the World Health Organization	5.250
	European Journal of Epidemiology	5.118
	American Journal of Epidemiology	4.780
	Cancer Epidemiology Biomarkers & Prevention	4.559
Radiology. Nuclear medicine and Medical imaging	Human Brain Mapping	6.878
	Radiology	6.339
	Neuroimage	6.252
	Jacc-Cardiovascular Imaging*	6.164
	Circulation-Cardiovascular Imaging	5.795
	Journal of Nuclear Medicine	5.774
	Investigative Radiology	5.460
	European Journal of Nuclear Medicine and Molecular Imaging	5.114
	International Journal of Radiation Oncology Biology Physics	4.524
	Radiotherapy and Oncology	4.520
Rehabilitation	Journal of Head Trauma Rehabilitation	4.443
	Neurorehabilitation and Neural Repair	4.278
	Ieee Transactions On Neural Systems and Rehabilitation Engineering	3.255
	Journal of Orthopaedic & Sports Physical therapy*	2.947
	Physical therapy*	2.778
	Supportive Care In Cancer	2.649
	Journal of Neuroengineering and Rehabilitation	2.567
	American Journal of Speech-Language Pathology	2.448
	Archives of Physical Medicine and Rehabilitation	2.358
	Journal of Physiotherapy	2.255
Reproductive biology	Human Reproduction Update*	8.847
	Human Reproduction*	4.670
	Molecular Human Reproduction	4.542
	Fertility and Sterility*	4.174
	Biology of Reproduction	4.027
	Reproduction	3.555
	American Journal of Reproductive Immunology	3.317
	Seminars In Reproductive Medicine*	3.211
	Reproductive Toxicology	3.141
	Placenta	3.117
Respiratory system	American Journal of Respiratory and Critical Care Medicine*	11.041

	Thorax	8.376
	European Respiratory Journal	6.355
	Chest*	5.854
	Journal of Heart and Lung Transplantation*	5.112
	Journal of Thoracic Oncology	4.473
	American Journal of Respiratory Cell and Molecular Biology	4.148
	Respiratory Research	3.642
	Journal of Thoracic and Cardiovascular Surgery	3.526
	American Journal of Physiology-Lung Cellular and Molecular Physiology	3.523
Rheumatology	Nature Reviews Rheumatology	9.745
	Annals of the Rheumatic Diseases	9.111
	Arthritis and Rheumatism	7.477
	Current Opinion In Rheumatology	5.191
	Arthritis Research & therapy	4.302
	Osteoarthritis and Cartilage*	4.262
	Rheumatology	4.212
	Seminars In Arthritis and Rheumatism	3.806
	Arthritis Care & Research	3.731
	Best Practice & Research In Clinical Rheumatology	3.550
Sport sciences	Exercise Immunology Review	7.053
	Exercise and Sport Sciences Reviews	5.283
	Sports Medicine	5.237
	Medicine and Science In Sports and Exercise	4.475
	American Journal of Sports Medicine*	4.439
	British Journal of Sports Medicine	3.668
	Journal of Applied Physiology	3.484
	Scandinavian Journal of Medicine & Science In Sports	3.214
	Journal of Orthopaedic & Sports Physical therapy*	2.947
	Journal of Science and Medicine In Sport	2.899
Surgery	Annals of Surgery	6.329
	American Journal of Transplantation*	6.192
	Endoscopy*	5.735
	Journal of Neurology Neurosurgery and Psychiatry	4.924
	American Journal of Surgical Pathology	4.868
	British Journal of Surgery	4.839
	Journal of the American College of Surgeons	4.500
	Surgery For Obesity and Related Diseases	4.121
	Annals of Surgical Oncology	4.120
	Archives of Surgery	4.100
Transplantation	American Journal of Transplantation*	6.192
	Journal of Heart and Lung Transplantation*	5.112
	Stem Cells and Development	4.670
	Cell Transplantation	4.422
	Liver Transplantation	3.944

	Biology of Blood and Marrow Transplantation	3.940
	Transplantation	3.781
	Bone Marrow Transplantation	3.541
	Nephrology Dialysis Transplantation	3.371
	Current Opinion In Organ Transplantation	3.272
Tropical medicine	Plos Neglected Tropical Diseases	4.569
	Malaria Journal	3.400
	Tropical Medicine & International Health	2.938
	Acta Tropica	2.787
	American Journal of Tropical Medicine and Hygiene	2.534
	Transactions of the Royal Society of Tropical Medicine and Hygiene	1.823
	Memorias Do Instituto Oswaldo Cruz	1.363
	Annals of Tropical Medicine and Parasitology	1.313
	Journal of Vector Borne Diseases	1.041
	Journal of Tropical Pediatrics	1.006
Urology & Nephrology	European Urology	10.476
	Journal of the American Society of Nephrology	8.987
	Nature Reviews Nephrology	7.943
	Kidney International	7.916
	American Journal of Kidney Diseases	5.294
	Clinical Journal of the American Society of Nephrology	5.068
	Nature Reviews Urology	4.793
	Current Opinion In Nephrology and Hypertension	3.964
	Prostate	3.843
	Journal of Urology	3.696
