# Supplementary material for: Poor reporting of multivariable prediction model studies: towards a targeted implementation strategy of the TRIPOD statement
Source: BMC Med. 2018 Jul 19;16:120. doi: 10.1186/s12916-018-1099-2 (PMC6052616; doi:10.1186/s12916-018-1099-2)
Supplement: Supplementary file 2 — Search strategy. (PDF 268 kb) [file 12916_2018_1099_MOESM2_ESM.pdf]

Search strategy

Pubmed search strategy on July 4th 2014

	hits
((Validat*[tiab] OR Predict*[ti] OR Rule*[tiab]) OR (Predict*[tiab] AND (Outcome*[tiab] OR Risk*[tiab] OR Model*[tiab])) OR ((History[tiab] OR Variable*[tiab] OR Criteria[tiab] OR Scor*[tiab] OR Characteristic*[tiab] OR Finding*[tiab] OR Factor*[tiab]) AND (Predict*[tiab] OR Model*[tiab] OR Decision*[tiab] OR Identif*[tiab] OR Prognos*[tiab])) OR (Decision*[tiab] AND (Model*[tiab] OR Clinical*[tiab] OR logistic models[mesh])) OR (Prognostic[tiab] AND (History[tiab] OR Variable*[tiab] OR Criteria[tiab] OR Scor*[tiab] OR Characteristic*[tiab] OR Finding*[tiab] OR Factor*[tiab] OR Model*[tiab]))) AND (0091-6749[is] OR 0105-4538[is] OR 1080-0549[is] OR 0954-7894[is] OR 1081-1206[is] OR 1528-4050[is] OR 0905-6157[is] OR 0105-1873[is] OR 1529-7322[is] OR 2092-7355[is] OR 0304-3959[is] OR 0003-3022[is] OR 0007-0912[is] OR 0003-2409[is] OR 1098-7339[is] OR 0003-2999[is] OR 1090-3801[is] OR 0375-9393[is] OR 0265-0215[is] OR 1530-7085[is] OR 0009-7322[is] OR 0195-668X[is] OR 0735-1097[is] OR 0009-7330[is] OR 1759-5002[is] OR 1942-325X[is] OR 1941-3289[is] OR 1936-8798[is] OR 1941-7640[is] OR 1936-878X[is] OR 1474-4422[is] OR 1759-4758[is] OR 1552-5260[is] OR 0364-5134[is] OR 0006-8950[is] OR 0001-6322[is] OR 1087-0792[is] OR 0028-3878[is] OR 0003-9942[is] OR 1522-8517[is] OR 1073-449X[is] OR 0090-3493[is] OR 0012-3692[is] OR 0342-4642[is] OR 1466-609X[is] OR 0897-7151[is] OR 0300-9572[is] OR 1541-6933[is] OR 1070-5295[is] OR 0375-9393[is] OR 0906-6713[is] OR 0022-0345[is] OR 1523-0899[is] OR 0109-5641[is] OR 0303-6979[is] OR 0905-7161[is] OR 0300-5712[is] OR 0099-2399[is] OR 1674-2818[is] OR 0266-4356[is] OR 0022-202X[is] OR 1755-1471[is] OR 0190-9622[is] OR 0003-987X[is] OR 0007-0963[is] OR 0906-6705[is] OR 0923-1811[is] OR 0001-5555[is] OR 0105-1873[is] OR 1660-5527[is] OR 0196-0644[is] OR 0300-9572[is] OR 1137-6821[is] OR 0022-5282[is] OR 0020-1383[is] OR 1090-3127[is] OR 1069-6563[is] OR 0735-6757[is] OR 1757-7241[is] OR 1472-0205[is] OR 0163-769X[is] OR 1550-4131[is] OR 1759-5029[is] OR 1043-2760[is] OR 0091-3022[is] OR 0012-1797[is] OR 0149-5992[is] OR 1083-3021[is] OR 0742-3098[is] OR 1523-0864[is] OR 0016-5085[is] OR 0270-9139[is] OR 0017-5749[is] OR 1759-5045[is] OR 0168-8278[is] OR 0272-8087[is] OR 0002-9270[is] OR 1542-3565[is] OR 0013-726X[is] OR 0016-5107[is] OR 0028-4793[is] OR 0140-6736[is] OR 0098-7484[is] OR 1756-1833[is] OR 1549-1676[is] OR 0003-4819[is] OR 0003-9926[is] OR 1741-7015[is] OR 0820-3946[is] OR 0954-6820[is] OR 0197-4580[is] OR 1568-1637[is] OR 1474-9718[is] OR 1525-8610[is] OR 1663-4365[is] OR 1079-5006[is] OR 1064-7481[is] OR 0161-9152[is] OR 0002-8614[is] OR 0531-5565[is] OR 0009-7330[is] OR 0887-6924[is] OR 0006-4971[is] OR 1066-5099[is] OR 1079-5642[is] OR 0340-6245[is] OR 1538-7933[is] OR 0268-960X[is] OR 0390-6078[is] OR 0271-678X[is] OR 0732-0582[is] OR 1474-1733[is] OR 1529-2908[is] OR 1074-7613[is] OR 0022-1007[is] OR 0105-2896[is] OR 0091-6749[is] OR 1471-4906[is] OR 1058-4838[is] OR 0952-7915[is] OR 1473-3099[is] OR 1058-4838[is] OR 0269-9370[is] OR 1080-6040[is] OR 0022-1899[is] OR 1560-7917[is] OR 0305-7453[is] OR 0951-7375[is] OR 1746-630X[is] OR 1525-4135[is] OR 1089-5159[is] OR 0944-7113[is] OR 0378-8741[is] OR 1534-7354[is] OR 0192-415X[is] OR 0965-2299[is] OR 1472-6882[is] OR 1741-427X[is] OR 0161-4754[is] OR 1075-5535[is] OR 0009-9147[is] OR 1040-8363[is] OR 0065-2423[is] OR 1931-5244[is] OR 1434-6621[is] OR 0009-8981[is] OR 0003-9985[is] OR 0009-9120[is] OR 0163-4356[is] OR 1552-4949[is] OR 1355-4786[is] OR 0029-7844[is] OR 0268-1161[is] OR 0015-0282[is] OR 0090-8258[is] OR 0002-9378[is] OR 1470-0328[is] OR 0960-7692[is] OR 1526-8004[is] OR 1072-3714[is] OR 0007-9235[is] OR 1474-175X[is] OR 1470-2045[is] OR 1535-6108[is] OR 0732-183X[is] OR 1759-4774[is] OR 0027-8874[is] OR 0887-6924[is] OR 2159-8274[is] OR 1350-9462[is] OR 0161-6420[is] OR 0003-9950[is] OR 0002-9394[is] OR 0146-0404[is] OR 0014-4835[is] OR 0039-6257[is] OR 0275-004X[is] OR 0007-1161[is] OR 1542-0124[is] OR 0363-5465[is] OR 1063-4584[is] OR 0021-9355[is] OR 1529-9430[is] OR 0749-8063[is] OR 0190-6011[is] OR 0736-0266[is] OR 0009-921X[is] OR 0031-9023[is] OR 1745-3674[is] OR 0196-0202[is] OR 1525-3961[is] OR 1043-3074[is] OR 0378-5955[is] OR 1420-3030[is] OR 1531-7129[is] OR 0023-852X[is] OR 0179-051X[is] OR 1749-4478[is] OR 0886-4470[is] OR 0890-8567[is] OR 0031-4005[is] OR 1072-4710[is] OR 0022-3476[is] OR 1018-8827[is] OR 0891-3668[is] OR 1744-165X[is] OR 1359-2998[is] OR 0905-6157[is] OR 0003-9888[is] OR 0009-7322[is] OR 0009-7330[is] OR 0194-911X[is] OR 1079-5642[is] OR 0039-2499[is] OR 0340-6245[is] OR 1538-7933[is] OR 0957-9672[is] OR 1567-5688[is] OR 0094-6176[is] OR 1544-1709[is] OR 1471-4418[is] OR 0960-1643[is] OR 0281-3432[is] OR 0263-2136[is] OR 0008-350X[is] OR 1557-2625[is] OR 0002-838X[is] OR 1471-2296[is] OR 1751-9918[is] OR 1359-4184[is] OR 0002-953X[is] OR 0003-990X[is] OR 0006-3223[is] OR 1723-8617[is] OR 0893-133X[is] OR 0586-7614[is] OR 0033-3190[is] OR 0890-8567[is] OR 0007-1250[is] OR 0193-936X[is] OR 0091-6765[is] OR 0300-5771[is] OR 0512-3054[is] OR 1044-3983[is] OR 0895-4356[is] OR 0042-9686[is] OR 0393-2990[is] OR 0002-9262[is] OR 1055-9965[is] OR 1065-9471[is] OR 0033-8419[is] OR 1053-8119[is] OR 1936-878X[is] OR 1941-9651[is] OR 0161-5505[is] OR 0020-9996[is] OR 1619-7070[is] OR 0360-3016[is] OR 0167-8140[is] OR 0885-9701[is] OR 1545-9683[is] OR 1534-4320[is] OR 0190-6011[is] OR 0031-9023[is] OR 0941-4355[is] OR 1743-0003[is] OR 1058-0360[is] OR 0003-9993[is] OR 1836-9553[is] OR 1355-4786[is] OR 0268-1161[is] OR 1360-9947[is] OR 0015-0282[is] OR 0006-3363[is] OR 1470-1626[is] OR 1046-7408[is] OR 1526-8004[is] OR 0890-6238[is] OR 0143-4004[is] OR 1073-449X[is] OR 0040-6376[is] OR 0903-1936[is] OR 0012-3692[is] OR 1053-2498[is] OR 1556-0864[is] OR 1044-1549[is] OR 1465-993X[is] OR 0022-5223[is] OR 1040-0605[is] OR 1759-4790[is] OR 0003-4967[is] OR 0004-3591[is] OR 1040-8711[is] OR 1478-6354[is] OR 1063-4584[is] OR 1462-0324[is] OR 0049-0172[is] OR 2151-464X[is] OR 1521-6942[is] OR 1077-5552[is] OR 0091-6331[is] OR 0112-1642[is] OR 0195-9131[is] OR 0363-5465[is] OR 0306-3674[is] OR 8750-7587[is] OR 0905-7188[is] OR 0190-6011[is] OR 1440-2440[is] OR 0003-4932[is] OR 1600-6135[is] OR 0013-726X[is] OR 0022-3050[is] OR 0147-5185[is] OR 0007-1323[is] OR 1072-7515[is] OR 1550-7289[is] OR 1068-9265[is] OR 0004-0010[is] OR 1600-6135[is] OR 1053-2498[is] OR 1547-3287[is] OR 0963-6897[is] OR 1527-6465[is] OR 1083-8791[is] OR 0041-1337[is] OR 0268-3369[is] OR 0931-0509[is] OR 1087-2418[is] OR 1935-2735[is] OR 1475-2875[is] OR 1360-2276[is] OR 0001-706X[is] OR 0002-9637[is] OR 0035-9203[is] OR 0074-0276[is] OR 0003-4983[is] OR 0972-9062[is] OR 0142-6338[is] OR 0302-2838[is] OR 1046-6673[is] OR 1759-5061[is] OR 0085-2538[is] OR 0272-6386[is] OR 1555-9041[is] OR 1759-4812[is] OR 1062-4821[is] OR 0270-4137[is] OR 0022-5347[is]) AND (2014/05/01 : 2014/06/01[dp])	4871
